# Bacterial Aspartyl-tRNA Synthetase Has Glutamyl-tRNA Synthetase Activity

**DOI:** 10.3390/genes10040262

**Published:** 2019-04-01

**Authors:** Udumbara M. Rathnayake, Tamara L. Hendrickson

**Affiliations:** Department of Chemistry, Wayne State University, 5101 Cass Avenue, Detroit, MI 48202, USA; udumbara.rathnayake@wayne.edu

**Keywords:** tRNA, misacylation, indirect tRNA aminoacylation, AspRS, GluRS-like

## Abstract

The aminoacyl-tRNA synthetases (aaRSs) are well established as the translators of the genetic code, because their products, the aminoacyl-tRNAs, read codons to translate messenger RNAs into proteins. Consequently, deleterious errors by the aaRSs can be transferred into the proteome via misacylated tRNAs. Nevertheless, many microorganisms use an indirect pathway to produce Asn-tRNA^Asn^ via Asp-tRNA^Asn^. This intermediate is produced by a non-discriminating aspartyl-tRNA synthetase (ND-AspRS) that has retained its ability to also generate Asp-tRNA^Asp^. Here we report the discovery that ND-AspRS and its discriminating counterpart, AspRS, are also capable of specifically producing Glu-tRNA^Glu^, without producing misacylated tRNAs like Glu-tRNA^Asn^, Glu-tRNA^Asp^, or Asp-tRNA^Glu^, thus maintaining the fidelity of the genetic code. Consequently, bacterial AspRSs have glutamyl-tRNA synthetase-like activity that does not contaminate the proteome via amino acid misincorporation.

## 1. Introduction

The fidelity of protein translation depends on the accurate pairing of cognate tRNAs to their cognate amino acids. The ligation of the amino acid to its tRNA is catalyzed by a highly specific group of enzymes known as the aminoacyl-tRNA synthetases (aaRSs) [1,2,3]. Under normal conditions, these enzymes maintain high accuracy and specificity in selecting their cognate amino acid and tRNA substrates. In fact, decades of research have been dedicated to demonstrating that the aaRSs are exquisitely specific for their cognate substrates and are key players in defining the accuracy of the proteome [2,4,5,6]. An increasing body of work, however, is emerging that demonstrates that some aaRSs can alter their activity or relax their selectivity in response to stress, even promoting errors in translation [7,8,9,10,11,12].

**Figure 1:**
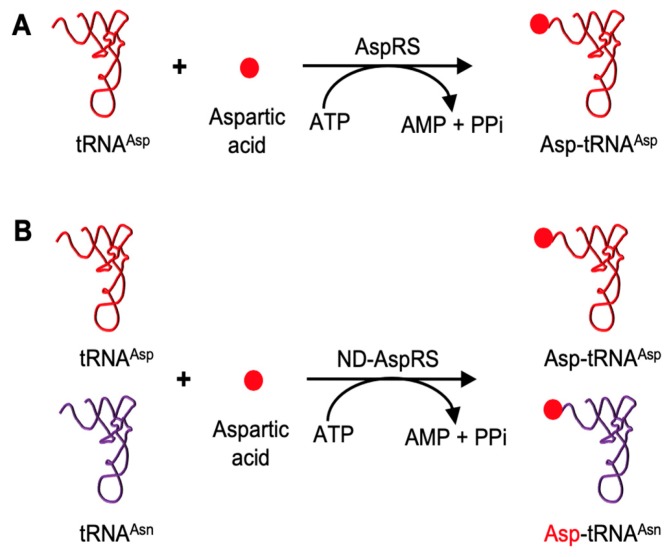
Canonical roles of AspRS and ND-AspRS. (**A**) AspRS aminoacylates tRNA^Asp^ with aspartic acid to produce Asp-tRNA^Asp^. (**B**) ND-AspRS catalyzes the aspartylation of both tRNA^Asp^ and tRNA^Asn^ to produce Asp-tRNA^Asp^ and the misacylated Asp-tRNA^Asn^.

Aspartyl-tRNA synthetase (AspRS) is an exception to the rule of one aaRS per amino acid/tRNA pair. This enzyme is found in two general forms, discriminating and non-discriminating, based on divergent tRNA specificities. Discriminating AspRS, the canonical enzyme, is found in eukaryotes, and some bacteria and archaea, and catalyzes the aspartylation of tRNA^Asp^ to produce Asp-tRNA^Asp^ (Figure 1A) [13]. The non-discriminating form, ND-AspRS, is found in many bacteria and archaea and some organelles; this enzyme cannot differentiate between tRNA^Asp^ and tRNA^Asn^ and aminoacylates both with aspartate to generate Asp-tRNA^Asp^ and the misacylated Asp-tRNA^Asn^, respectively (Figure 1B) [14,15,16,17]. To maintain the fidelity of the genetic code, Asp-tRNA^Asn^ is then converted to Asn-tRNA^Asn^ by a glutamine-dependent amidotransferase (AdT) [14,15,18,19].

The requirement for a discriminating AspRS versus an ND-AspRS differs from organism to organism; however, ND-AspRS is always accompanied by AdT [15,18,19,20]. ND-AspRS and AdT are typically found in organisms that lack asparaginyl-tRNA synthetase (AsnRS) and/or asparagine synthetase (AsnS). For example, *Pseudomonas aeruginosa* and *Helicobacter pylori* (*H. pylori* or *Hp*) lack both AsnRS and AsnS and consequently require ND-AspRS and AdT to produce both Asn-tRNA^Asn^ and asparagine [21,22]. *Mycobacterium tuberculosis* lacks AsnRS but has AsnS, so it only uses indirect tRNA aminoacylation to produce Asn-tRNA^Asn^ [12]. In contrast, *Staphylococcal aureus* (*S. aureus* or *Sa*) has a functioning AsnRS but lacks AsnS; thus, indirect aminoacylation is still required as the sole biosynthetic route to asparagine [23]. Some bacteria like *Thermus thermophilus* and *Deinococcus radiodurans* encode for more than one copy of AspRS [24,25]. In these cases, the longer, bacterial-type AspRS is discriminating and catalyzes the synthesis of Asp-tRNA^Asp^. The second AspRS (AspRS2) is non-discriminating and produces Asp-tRNA^Asn^ along with Asp-tRNA^Asp^.

While ND-AspRS clearly demonstrates relaxed tRNA substrate specificity, it remains fine-tuned as it only aspartylates tRNA^Asp^ and tRNA^Asn^, only in organisms that have AdT, and it presumably discriminates against other tRNAs as potential substrates. In this way, the fidelity of the genetic code is maintained. Evidence of further relaxation in substrate specificity for either a discriminating AspRS or an ND-AspRS has not been reported to our knowledge. Here we demonstrate that these enzymes are capable of specifically producing Glu-tRNA^Glu^ while maintaining their canonical roles: The production of Asp-tRNA^Asp^ (AspRS and ND-AspRS) and Asp-tRNA^Asn^ (ND-AspRS only). To the best of our knowledge, this is the first example of an aaRS capable of producing a non-cognate, but correctly aminoacylated tRNA.

## 2. Materials and Methods

### 2.1. Materials

Potassium dihydrogen phosphate (KH_2_PO_4_), sodium dihydrogen phosphate (NaH_2_PO_4_), L-aspartic acid (catalog #A93100, batch #12002LC), L-glutamic acid (catalog #G1251, lot #SLBK8671V), magnesium chloride, and oligonucleotides were purchased from Sigma-Aldrich (St. Louis, MO, USA). The radiolabeled reagents aspartic acid, L-[2,3-3H] (catalog #ART 0211, lot #180606), glutamic acid, L-[2,3,4-3H] (catalog #ART 0132, lot #180710) and adenosine triphosphate (ATP) [γ-^32^P] were purchased from American Radiolabeled Chemicals (St. Louis, MO, USA). Ampicillin, chloramphenicol, kanamycin, 4-(2-hydroxyethyl)-1-piperazineethanesulfonic acid (HEPES), ethylenediaminetetraacetic acid (EDTA), isopropyl β-d-thiogalactopyranoside (IPTG), phenylmethanesulfonyl fluoride (PMSF), and tris(hydroxymethyl) aminomethane (Tris) were from GoldBio Biotechnology, Inc., (St. Louis, MO, USA). Potassium chloride (KCl), sodium chloride (NaCl), trichloroacetic acid (TCA), and ammonium acetate (NH_4_OAc) were purchased from Fisher Scientific (Hampton, NH, USA).

### 2.2. Overexpression and Purification of aaRSs

*Hp* ND-AspRS [22], the *Hp* tRNA^Gln^-specific glutamyl-tRNA synthetase (GluRS2) [26], *Mycobacterium smegmatis* (*M. smegmatis* or *Ms*) ND-AspRS (vector provided by Dr. Babak Javid), and *Escherichia coli* (*E. coli* or *Ec*) AspRS and GluRS (vectors provided by Dr. Takuya Ueda [27]) were each overexpressed in *Ec* Bl21 (DE3) RIL in Luria-Bertani medium (LB, 1 L) inoculated with a saturated overnight culture grown from a single colony. The cultures were incubated at 37 °C, 200 rpm. IPTG (1 mM) was used to induce overexpression in each case. For *Hp* ND-AspRS and GluRS2 overexpression, the medium was supplemented with ampicillin (100 μg/mL), chloramphenicol (100 μg/mL), and glucose (0.5%). IPTG was added when the OD_600_ reached 0.8–1.0. Cells were collected after a 1 h induction period. For *Ms* ND-AspRS, the only difference was that kanamycin (25 μg/mL) was added instead of ampicillin. The two discriminating *Ec* enzymes were grown in the same medium as the two *Hp* enzymes. IPTG induction was initiated when the OD_600_ reached between 0.4 and 0.6 and continued for 4 h. Cells were harvested by centrifugation and frozen at −80 °C until ready for use.

The *Sa* ND-AspRS (vector provided by Dr. Kelly Sheppard) was overexpressed and purified as previously described [23]. In all other cases, cell pellets were suspended in lysis buffer (50 mM NaH_2_PO_4_, pH 7.4, 300 mM NaCl, 10 mM imidazole) and the cells were lysed with lysozyme (2 mg/mL) and sonication. Saturated PMSF (15 μL/mL) was added every 20 min to reduce proteolysis. Typically, a cell extract was added to a polyprep column that contained high-density cobalt agarose beads (~2 mL, Gold Biotechnology, Inc.) pre-washed with lysis buffer. The lysate was incubated with the resin by rotating at 4 °C for 1 h. The His_6_-tagged aaRSs were eluted according to the manufacturer’s protocol.

The eluents were dialyzed against dialysis buffer (20 mM KH_2_PO_4_, pH 7.2, 0.5 mM Na_2_EDTA, 5 mM β-mercaptoethanol) at 4 °C for 1–2 h and then against fresh dialysis buffer overnight. The aaRSs were adsorbed onto a HiTrap^TM^ DEAE FF column (GE Healthcare Life Sciences, Pittsburgh, PA, USA). The proteins were eluted with a stepwise gradient of 20–300 mM KH_2_PO_4_, pH 7.2, supplemented with 0.5 mM Na_2_EDTA, 5 mM β-mercaptoethanol. The proteins were concentrated with 30 kDa molecular weight spin filters (EMD Millipore, Burlington, MA, USA). After this two-column purification procedure, all enzymes were judged to have been purified to near homogeneity by SDS-PAGE (Figure S1). Final protein concentrations were determined by UV-Vis spectroscopy at 280 nm, using extinction coefficients calculated using the ExPASy ProtParam tool [28]. The aaRSs were immediately used in aminoacylation assays. All experiments were conducted using biological replicates in triplicate.

### 2.3. In Vivo Transcription and Purification of tRNAs

*Hp* tRNA^Asn^ [22] and tRNA^Gln^ [26] were overexpressed in *Ec* MV1184 and purified as previously described [29]. After deacylation, the tRNAs were electroeluted in a denaturing urea gel for further purification. The tRNA band was excised from the gel, crushed, and incubated overnight at 37 °C in crush and soak buffer (0.5 mM NH_4_OAc, pH 5.2, 1 mM Na_2_EDTA). The eluted tRNA sample was isopropanol precipitated. The resultant pellet was dissolved in water, folded, and quantified by aminoacylation assay, as previously described [29]. These purifications yield tRNA that is enriched with the overexpressed tRNA isoacceptor but also contains total *Ec* tRNA. Separately, total *Ec* tRNA was isolated from a saturated *Ec* MV1184 culture that had been grown at 37 °C in LB medium supplemented with glucose (0.5%) and purified as described [29]. The concentration of total *Ec* tRNA was calculated by UV-Vis spectroscopy at 260 nm assuming 1 OD = 1.6 μM.

### 2.4. Initial Rate Aminoacylation Assays

Each tRNA was pre-folded as previously described [29]. Aminoacylation assays were conducted in buffer containing 100 mM Na-HEPES, pH 7.5, 30 mM KCl, 12 mM MgCl_2_, 2 mM ATP, 0.1 mg/mL BSA, 20 μM amino acid, 50 μCi/mL ^3^H-labeled amino acid, and 10 μM *Hp* tRNA^Asn^ or tRNA^Gln^ as noted. Assays were conducted at 37 °C and were initiated with the addition of 200 nM *Hp* ND-AspRS or GluRS2. Aliquots were removed and quenched on Whatman filter pads containing TCA. The pads were washed 3 times for 15 min with chilled 5% TCA. Pads were dried and counted in 3 mL ECOLITE (+) scintillation fluid (MP Biomedicals, Solon, OH, USA). Aminoacylation assays with total *Ec* tRNA were carried out in the same buffer containing 50–100 μM total tRNA. *Hp* ND-AspRS (100 nM or 500 nM) or *Hp* GluRS2 (100 nM or 500 nM) were used in these assays as noted.

### 2.5. Extended Aminoacylation Assays (90 Min)

*Hp* ND-AspRS and GluRS2 used in these assays were only purified by cobalt affinity purification. The aminoacylation assays were conducted at 37 °C in buffer containing 20 mM HEPES-OH, pH 7.5, 4 mM MgCl_2_, 2 mM ATP, 100 μM amino acid, and 25 μCi/mL ^3^H-labeled amino acid. All aaRSs were added to a final concentration of 1 μM and overexpressed tRNA isoacceptor was added to a final concentration of 10 μM.

### 2.6. Acid Gel Electrophoresis and Northern Blot Analysis

Aminoacylation reactions were conducted in the same buffer used for initial rate aminoacylation assays only without radiolabeled amino acid for 90 min at 37 °C with 500 nM aaRS. NaOAc (0.3 mM, pH 4.5) was added to each reaction and they were quenched with phenol/chloroform (1:1 *v*/*v*, pH 4.5). The tRNAs were ethanol precipitated and the resultant pellets were dissolved in buffer containing 10 mM NaOAc, pH 4.5, 1 mM Na_2_EDTA. Acid gel electrophoresis was used to separate deacylated tRNAs from their aminoacylated counterparts. The tRNA samples were quantified by UV-Vis spectroscopy at 260 nm, and 0.05 OD_260_ units were loaded per lane for overexpressed tRNAs. This amount was increased to 0.25 OD_260_ units for total *Ec* tRNA. Isoacceptor-specific oligonucleotides were used in Northern blots to visualize each tRNA (Table S1). The tRNA specific DNA probes were prepared as previously described [26]. The acid gel and Northern blot analyses were performed as previously described [26,30], with the exception that a non-specific DNA oligonucleotide (0.1 μM) was added during the first three washing steps after probe hybridization.

## 3. Results

### 3.1. H. pylori ND-AspRS Appears to Aminoacylate H. pylori tRNA^Gln^ with Aspartate and Glutamate

**Figure 2:**
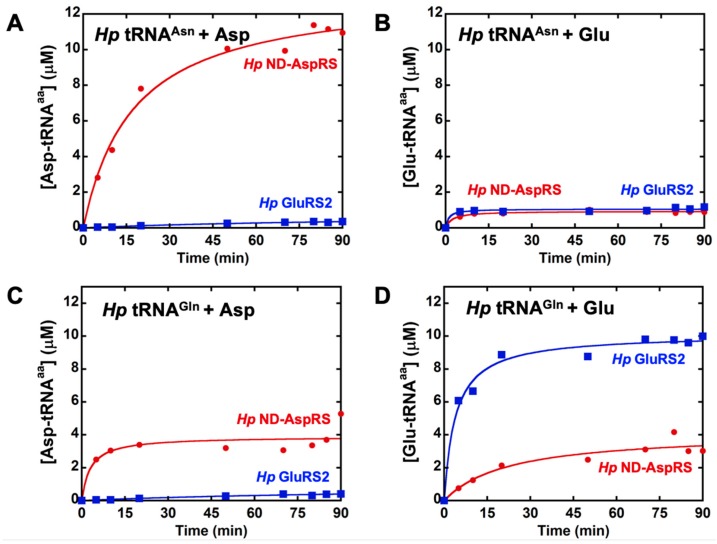
*H. pylori* ND-AspRS appears to attach aspartate and glutamate to tRNA^Gln^. *Hp* ND-AspRS (●, 1 μM) and GluRS2 (■, 1 μM) were tested in cross-aminoacylation assays using *Hp* tRNA^Asn^ and tRNA^Gln^ with aspartate and glutamate. The tRNA isoacceptor concentration in each assay was 10 μM; each tRNA was contaminated with total *Ec* tRNA. (**A**) *Hp* tRNA^Asn^ aminoacylated with aspartate, (**B**) *Hp* tRNA^Asn^ aminoacylated with glutamate, (**C**) *Hp* tRNA^Gln^ aminoacylated with aspartate, and (**D**) *Hp* tRNA^Gln^ aminoacylated with glutamate.

ND-AspRS has relaxed tRNA specificity and aspartylates both tRNA^Asp^ and tRNA^Asn^ to produce Asp-tRNA^Asp^ and Asp-tRNA^Asn^, respectively [14,15]. We have previously demonstrated that the *Hp* ND-AspRS has this dual tRNA specificity, as expected [22]. Given the nature of the genetic code and decades of work characterizing the aaRSs, ND-AspRS should not demonstrate broader substrate specificity for non-cognate tRNAs or amino acids. We examined *Hp* ND-AspRS and GluRS2, a tRNA^Gln^-specific GluRS [26,31], for possible cross-reactivity with overexpressed tRNA^Asn^ and tRNA^Gln^ with both aspartate and glutamate (Figure 2). We performed these assays with longer time points to look at plateau levels of aminoacylation and to detect any low levels of aminoacylation. As expected, ND-AspRS produced Asp-tRNA^Asn^ and did not produce Glu-tRNA^Asn^ (Figure 2A,B, respectively). Unexpectedly, this enzyme showed aminoacylation activity in both assays with tRNA^Gln^ (Figure 2C,D), suggesting that it was producing the non-cognate product *Hp* Asp-tRNA^Gln^ and Glu-tRNA^Gln^. However, because these tRNAs were overexpressed in vivo, a practice necessary to introduce required post-transcriptional modifications like queuosine [32] and 2-thiouridine [33], the tRNAs used in these experiments were contaminated with total *Ec* tRNA. Thus, the possibility also exists that *Hp* ND-AspRS is aminoacylating one or more *Ec* tRNAs instead. For comparison, GluRS2 showed only its expected activity, producing Glu-tRNA^Gln^ (Figure 2D), but not Asp-tRNA^Asn^, Glu-tRNA^Asn^, or Asp-tRNA^Gln^ (Figure 2A–C). We conducted these same, longer aminoacylation assays using the *Ms* ND-AspRS and ND-GluRS (Figure S2): We observed the same behavior with glutamate, indicating that this phenomenon is found in bacteria beyond *H. pylori.*

We repeated the assays shown in Figure 2 with shorter time points and lower enzyme concentrations to look at initial rates (Figure S3). Under these more standard conditions, the ability of ND-AspRS to ligate glutamate onto either *Hp* tRNA^Gln^ or a contaminating *Ec* tRNA was masked (Figure S3D), offering an explanation for why this activity has remained undiscovered. Robust aspartylation by *Hp* ND-AspRS, presumably of contaminating *Ec* tRNA^Asp^ and/or tRNA^Asn^_,_ was observed as anticipated (Figure S3C).

Our use of extended time point assays (Figure 2) revealed an unexpected activity for ND-AspRS that was invisible under initial rate conditions (Figure S3). To our knowledge, this is the first evidence of an ND-AspRS utilizing glutamate instead of aspartate as its amino acid substrate. This activity makes some sense, however, given the close structural similarities between aspartate and glutamate. Nevertheless, the results presented in Figure 2 and Figures S2 and S3, still contained ambiguity with respect to the identity of the relevant tRNA substrate since each tRNA was overexpressed and purified with contaminating *Ec* tRNAs.

### 3.2. H. pylori ND-AspRS Aminoacylates E. coli tRNA^Glu^ with Glutamate to Produce Glu-tRNA^Glu^

To more precisely examine the unexpected aminoacylation reaction(s) catalyzed by *Hp* ND-AspRS, we turned to acid gel electrophoresis and Northern blotting techniques [30]. We continued to use *Hp* GluRS2 as a control because of its high specificity for Glu-tRNA^Gln^ production [26,31]. The advantages of this approach are two-fold. First, the acidic pH of these gels enables the separation of aminoacyl-tRNAs from their deacylated counterparts due to the extra charge provided by the protonated amino terminus of the amino acid (30). Second, oligonucleotides can be designed for Northern blot visualizations that are specific for a desired tRNA isoacceptor. Thus, this approach allows us to unequivocally identify the tRNA(s) being aminoacylated in a given experiment, clearly resolving the ambiguity of the results presented above. Since *Hp* ND-AspRS uses glutamate as a substrate (Figure 2D), we hypothesized that it may be adding glutamate onto *Hp* tRNA^Gln^ or *Ec* tRNA^Glu^. Consequently, we used this methodology to examine *Hp* ND-AspRS-catalyzed aminoacylation of *Hp* tRNA^Gln^ and *Ec* tRNA^Glu^ with aspartate versus glutamate as its amino acid substrates. Northern blots were conducted with overexpressed *Hp* tRNA^Gln^ contaminated with total *Ec* tRNA as a source of *Ec* tRNA^Glu^. In both cases, oligonucleotides were designed to visualize only the tRNAs of interest: *Hp* tRNA^Gln^ and *Ec* tRNA^Glu^.

**Figure 3:**
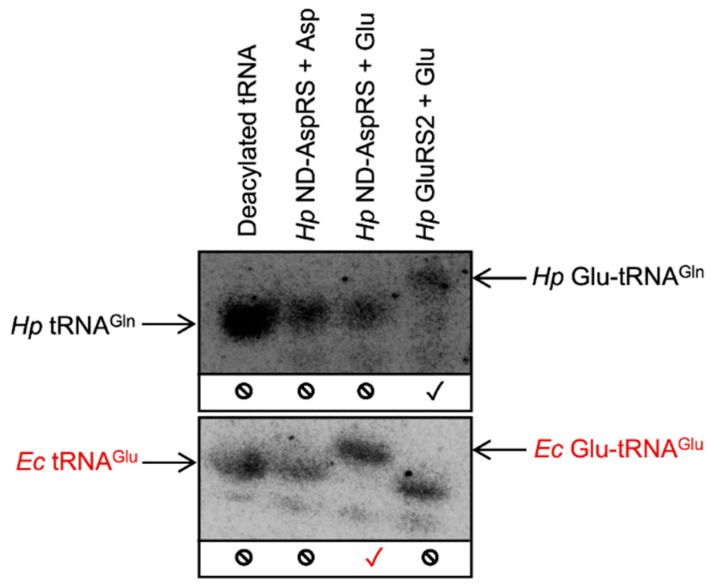
*H. pylori* ND-AspRS aminoacylates *E. coli* tRNA^Glu^ with glutamate producing Glu-tRNA^Glu^. *Hp* tRNA^Gln^, contaminated with total *Ec* tRNA, was aminoacylated with either *Hp* ND-AspRS or GluRS2 and with aspartate versus glutamate. Aminoacylated versus deacylated tRNAs were separated in an acid gel. Specific tRNAs and aminoacyl-tRNAs were imaged using ^32^P-labeled oligonucleotides in Northern blots. The blots were visualized with a *Hp* tRNA^Gln^-specific primer (blot 1) and an *Ec* tRNA^Glu^-specific primer (blot 2). Expected tRNA aminoacylation activity is indicated with a black check mark (✓); unexpected aminoacylation activity is indicated in red (✓); the absence of aminoacylation activity with a given tRNA is indicated with a no symbol (⦸).

*Hp* ND-AspRS does not aminoacylate *Hp* tRNA^Gln^ with either aspartate or glutamate (Figure 3, blot 1, lanes 2 and 3), suggesting that the activities observed above (Figure 2C,D) are the result of aminoacylation of one or more *Ec* tRNAs (compared to the production of Glu-tRNA^Gln^ by *Hp* GluRS2 in Figure 3, blot 1, lane 4.) In contrast, *Hp* ND-AspRS aminoacylates *Ec* tRNA^Glu^ with glutamate to produce Glu-tRNA^Glu^ (Figure 3, blot 2, lane 3), offering direct evidence that this ND-AspRS has non-canonical aminoacylation activity that goes beyond its ability to aspartylate tRNA^Asp^ and tRNA^Asn^. We considered the possibility that *E. coli* glutamyl-tRNA synthetase (*Ec* GluRS) had inadvertently co-purified with *Hp* ND-AspRS, however, no evidence of this contamination was observed by SDS-PAGE (Figure S1). Remarkably, *Hp* ND-AspRS remains accurate by pairing glutamate with tRNA^Glu^ to produce correctly acylated Glu-tRNA^Glu^ but not the misacylated Asp-tRNA^Glu^ (Figure 3, blot 2, lanes 3 and 2 respectively). In other words, this enzyme is exhibiting activity consistent with a canonical GluRS, in that it is ligating glutamate specifically to tRNA^Glu^. 

**Figure 4:**
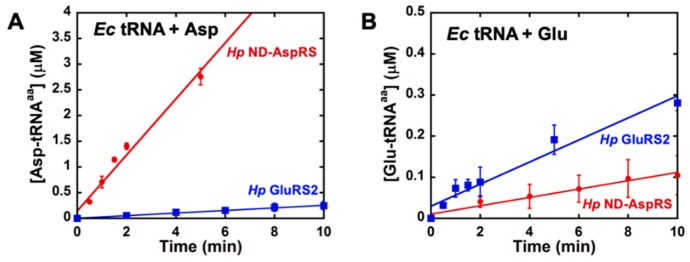
*H. pylori* ND-AspRS aminoacylates *E. coli* tRNA with glutamate. *Hp* ND-AspRS was tested for its activity to aminoacylate *Ec* tRNA (50–100 μM) with aspartate and glutamate. *Hp* GluRS2 was also assayed for comparison. (**A**) Aminoacylation of *Ec* tRNA with aspartate by *Hp* ND-AspRS (●, 100 nM) versus GluRS2 (■, 500 nM). (**B**) Aminoacylation of *Ec* tRNA with glutamate by *Hp* ND-AspRS (●, 500 nM) versus GluRS2 (■, 100 nM). Error bars represent standard deviation from biological replicates in triplicate.

Next, we compared the initial rates and extents of glutamylation of total *Ec* tRNA by *Hp* GluRS2, an enzyme that naturally uses glutamate as its substrate [26,31], and *Hp* ND-AspRS, which unexpectedly also uses glutamate (Figure 4 and Figure S4). Here, to ensure that we could visualize this novel activity, we increased the concentration of each enzyme when looking at aminoacylation of a non-cognate tRNA. We also verified that *Ms* ND-AspRS has this ability to glutamylate total *Ec* tRNA (Figure S5). In these experiments, the ability of ND-AspRS to produce Glu-tRNA^Glu^ is clearly apparent.

### 3.3. Glu-tRNA^Glu^ Production Is Common among Bacterial Non-Discriminating and Discriminating AspRSs

**Figure 5:**
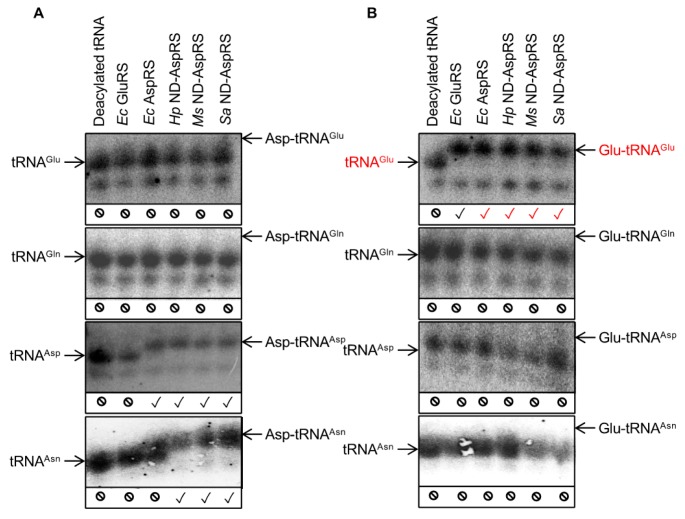
Some bacterial AspRSs aminoacylate *E. coli* tRNA^Glu^ with glutamate producing Glu-tRNA^Glu^. Total *Ec* tRNA was aminoacylated with (**A**) aspartate and (**B**) glutamate by *Ec* GluRS, *Ec* AspRS, *Hp* ND-AspRS, *Ms* ND-AspRS, and *Sa* ND-AspRS. The aminoacylated versus deacylated tRNAs were separated in acid gels. ^32^P-labeled oligonucleotides specific for *Ec* tRNA^Glu^, tRNA^Gln^, tRNA^Asp^, and tRNA^Asn^ were used for each blot as indicated. Expected tRNA aminoacylation activities are indicated with a black check mark (✓); unexpected activities are indicated in red (✓); the absence of aminoacylation activity with a given tRNA is indicated with a no symbol (⦸).

All experiments described so far with *Hp* and *Ms* ND-AspRS demonstrate that these enzymes can attach glutamate to *Ec* tRNA and *Hp* ND-AspRS specifically produces *Ec* Glu-tRNA^Glu^. Given that these are inter-species pairings in both cases, we decided that it was important to examine the *Ec* discriminating AspRS for this activity with its homologous *Ec* tRNA. We also included the ND-AspRS from *S. aureus* to gain additional perspective into the ubiquity of this unexpected activity. Acid gels and Northern blot analyses using oligonucleotide probes that are specific for four different *Ec* tRNAs (tRNA^Glu^, tRNA^Gln^, tRNA^Asp^, and tRNA^Asn^) were conducted with all four enzymes: The discriminating AspRS from *Ec* and the ND-AspRSs from *Hp, Ms,* and *Sa* (Figure 5). In many of the Northern blots shown below, the deacylated and the aminoacylated tRNAs appear as two bands, presumably due to differences in post-transcriptional modifications of the tRNAs, as previously reported [26].

Figure 5 shows the Northern blot results obtained from total *Ec* tRNA aminoacylated with aspartate (A) and glutamate (B) by different bacterial aaRSs: *Ec* GluRS was used as a control. The results of panel A reveal that each AspRS attaches aspartate to tRNA^Asp^ and the three ND-AspRSs also attach aspartate to tRNA^Asn^, as expected (Figure 5A, blots 3 and 4). Furthermore, none of the enzymes tested were capable of attaching significant amounts of aspartate to either non-cognate tRNA substrates tRNA^Glu^ or tRNA^Gln^ (Figure 5A, blots 1 and 2).

The most interesting and critical results are revealed in blot 1 in Figure 5B and are highlighted with red checkmarks. This blot was probed with a ^32^P-labeled oligonucleotide specific for *Ec* tRNA^Glu^ and Glu-tRNA^Glu^ formation with *Ec* GluRS was used as a positive control. A clear shift is observed between deacylated tRNA^Glu^ and Glu-tRNA^Glu^ produced by *Ec* GluRS (Figure 5B, blot 1, lanes 1 and 2). What is remarkable is that all four AspRSs showed this same shift, clearly indicative of ubiquitous Glu-tRNA^Glu^ production. The remaining blots in Figure 5B demonstrate that this glutamylation activity is specific for tRNA^Glu^ as none of the enzymes tested were capable of adding glutamate onto *Ec* tRNA^Gln^, tRNA^Asp^, or tRNA^Asn^. These data demonstrate that *Ec* AspRS and the ND-AspRSs from *Hp, Ms*, and *Sa* aminoacylate tRNA^Glu^ with glutamate to produce Glu-tRNA^Glu^. These reactions represent a new, non-canonical activity for AspRS that can be viewed as that of a discriminating GluRS.

## 4. Discussion

In this work, we demonstrate that bacterial discriminating and non-discriminating AspRSs have quantifiable GluRS activity as they are capable of specifically generating Glu-tRNA^Glu^. Using total *Ec* tRNA, this activity was observed with four different AspRSs: The *Ec* discriminating AspRS and three non-discriminating AspRSs from *Hp, Ms,* and *Sa.* All four enzymes have canonical AspRS activity and produce Asp-tRNA^Asp^ (Figure 5A, blot 3). The three ND-AspRSs also produce misacylated Asp-tRNA^Asn^ (Figure 5A, blot 4); this intermediate would be converted to Asn-tRNA^Asn^ in vivo by AdT. All four enzymes were also capable of specifically producing Glu-tRNA^Glu^ (Figure 5B, blot 1) without adding glutamate to other related tRNAs.

We considered the possibility of the co-purification of contaminating *Ec* GluRS as an alternative explanation for this activity. However, each AspRS was purified in two steps: cobalt affinity and DEAE chromatography. *Ec* GluRS elutes from our DEAE column at a lower KH_2_PO_4_ concentration (~50–100 mM) than the tested AspRSs (~200–300 mM), such that any GluRS that survived the affinity purification step would have been removed by DEAE chromatography. Furthermore, no evidence of *Ec* GluRS contamination was visible by using SDS-PAGE gel (Figure S1). Thus, this two-step purification strategy eliminates the possibility of contaminated *Ec* GluRS in the AspRS samples, confirming that this activity is due to AspRS alone.

This GluRS-like activity of AspRSs is remarkable and unexpected because it is accurate and yet outside the normal purview of an AspRS. It truly is GluRS activity as each AspRS specifically attaches glutamate only to tRNA^Glu^ producing Glu-tRNA^Glu^. The fact that *Ec* AspRS demonstrates this activity is particularly important because of the homologous nature of this result: *Ec* AspRS produces *Ec* Glu-tRNA^Glu^. All other results herein arose from heterologous pairings of an ND-AspRS with *Ec* tRNA. Our results with *Ec* AspRS demonstrate that this activity is not simply a result of cross-species recognition of a non-cognate tRNA. It is also remarkable that no evidence of misacylated Asp-tRNA^Glu^, Glu-tRNA^Asp^, or Glu-tRNA^Asn^ was observed, given the ability of these AspRSs to produce Glu-tRNA^Glu^. This observation is important because it demonstrates that this GluRS-like activity does not threaten the fidelity of the genetic code. Nevertheless, it poses an unexpected problem with molecular recognition. How does AspRS know to specifically attach aspartate to tRNA^Asp^ (and tRNA^Asn^) and glutamate to tRNA^Glu^ without cross misacylation of these tRNAs? Our results suggest that communication between the amino acid and tRNA binding sites must occur to achieve this specificity. Further research is needed to understand this apparent tRNA-induced conformational change.

AaRSs almost always recognize discrete molecular features in their tRNA substrates, termed identity elements. These determinants can be positive and favor recognition or negative and disfavor interactions. For *Ec* tRNA^Asp^, the GUC anticodon, the G73 discriminator base, the G2:C71 base pair in the acceptor stem, C38 in the anticodon loop, and G10 in the D arm of the tRNA serve as the positive identity elements for *Ec* AspRS [34,35,36]. In contrast, *Ec* GluRS recognizes the G1:C72 and U2:A71 base pairs in the acceptor stem, A37 in the anticodon loop, U11:A24 base pair in D arm, and the U13:G22-A46 tertiary base pair in *Ec* tRNA^Glu^. Further, the lack of a nucleotide at position 47 and the modified uridine (s^2^U) in the UUC anticodon of tRNA^Glu^ are also known identity determinants [33,34,37,38,39]. *Ec* tRNA^Glu^ does contain several tRNA^Asp^ identity elements: The G73 discriminator base, G10 in the D arm, and C38 in the anticodon loop. But are these shared elements enough for recognition of *Ec* tRNA^Glu^ by *Ec* AspRS? Or is an alternative, expanded identity set recognized in such a way as to favor glutamylation of *Ec* tRNA^Glu^? To answer these questions, the identity elements in *Ec* tRNA^Glu^ that allow recognition by *Ec* AspRS would have to be specifically evaluated.

In conclusion, we have demonstrated that bacterial AspRSs have GluRS activity and are capable of producing Glu-tRNA^Glu^, at least in vitro. The relevance of this reaction in vivo is very difficult to demonstrate as it is unlikely that this activity is robust enough to enable deletion of the native *Ec* GluRS, especially in the presence of the cognate tRNA^Asp^ substrate. It is possible that this activity exists as a backup in case the cognate GluRS becomes damaged, for example. It is also possible that it is a remnant of early AspRS evolution. The early genetic code was likely sloppy with ancestral aaRSs capable of aminoacylating multiple tRNAs with different amino acids [5]. Evidence also suggests that Class I and II enzymes emerged in pairs [40,41], with early AspRSs and GluRSs perhaps as a co-evolving pair that recognized and acylated the same early tRNA or tRNA-like substrates. These enzymes would have recognized opposite faces of the tRNA, facilitating this co-evolution and protecting the tRNA from hydrolysis [41]. Given that the background GluRS activity exhibited by AspRS does not put the genetic code in jeopardy, it would not have been selected against as the genetic code evolved to be more selective and specific. The results presented here also suggest the possibility that other aaRSs retain similar background activities, a hypothesis that awaits further testing.

## Supplementary Materials

The following are available online at https://www.mdpi.com/2073-4425/10/4/262/s1, Methodology for the overexpression and purification of *Ms* ND-GluRS, in vitro transcription of tRNAs, and extending tRNA aminoacylation assays. Figure S1. SDS-PAGE gels of purified aaRSs. Figure S2. Extended *M. smegmatis* tRNA^Asn^ and tRNA^Gln^ aminoacylation assays with *M. smegmatis* ND-AspRS and ND-GluRS with aspartate versus glutamate. Figure S3. *H. pylori* ND-AspRS shows unexpected aminoacylation activity with overexpressed *H. pylori* tRNA^Gln^. Figure S4. Extended total *E. coli* tRNA aminoacylation assays with *H. pylori* ND-AspRS and GluRS2 with aspartate versus glutamate. Figure S5. Extended total *E. coli* tRNA aminoacylation assays with *M. smegmatis* ND-AspRS and ND-GluRS with aspartate versus glutamate. Table S1: The tRNA specific oligonucleotide sequences used in northern blot analysis
