# Supplementary material for: Bacterial Aspartyl-tRNA Synthetase Has Glutamyl-tRNA Synthetase Activity
Source: Genes (Basel). 2019 Apr 1;10(4):262. doi: 10.3390/genes10040262 (PMC6523644; doi:10.3390/genes10040262)
Supplement: Supplementary file 1 [file genes-10-00262-s001.pdf]

**Supplementary Data**

**for**

# Bacterial Aspartyl-tRNA Synthetase Has Glutamyl-tRNA Synthetase Activity

Udumbara M. Rathnayake and Tamara L. Hendrickson*

Department of Chemistry, Wayne State University, 5101 Cass Avenue, Detroit, MI 48202

*To Whom Correspondence Should be Addressed:

Email: Tamara.Hendrickson@wayne.edu

Phone: 313-577-6914

## Overexpression and purification of *M. smegmatis* ND-GluRS

The *Ms* ND-GluRS (vector provided by Dr. Babak Javid) was also overexpressed in *Ec* Bl21(DE3) RIL in LB medium supplemented with kanamycin (25 μg/mL), chloramphenicol (100 μg/mL) and glucose (0.5%). Cultures were grown at 37 °C to an $OD_{600}$ of 0.8-1.0 and induced with IPTG (1 mM) for one hour. The protein was purified using the same purification method as the other aaRSs (affinity chromatography and DEAE).

## *In vivo* transcription and purification of *M. smegmatis* tRNAs

*Ms* $tRNA^{Asn}$ and $tRNA^{Gln}$ (vectors provided by Dr. Babak Javid) were overexpressed in *Ec* MV1184 and purified as described in the main text.

## Extended aminoacylation assays (90 min) by aaRSs

All the aaRSs used in these assays were only purified by cobalt affinity purification. The aminoacylation assays were conducted in buffer containing 20 mM HEPES-OH, pH 7.5, 4 mM $MgCl_2$, 2 mM ATP, 100 μM amino acid, and 25 μCi/mL $^3$H labeled amino acid. All aaRSs were added to a final concentration of 1 μM. Overexpressed tRNA isoacceptor (10 μM) or total *Ec* tRNA (50-100 μM) were used as indicated.

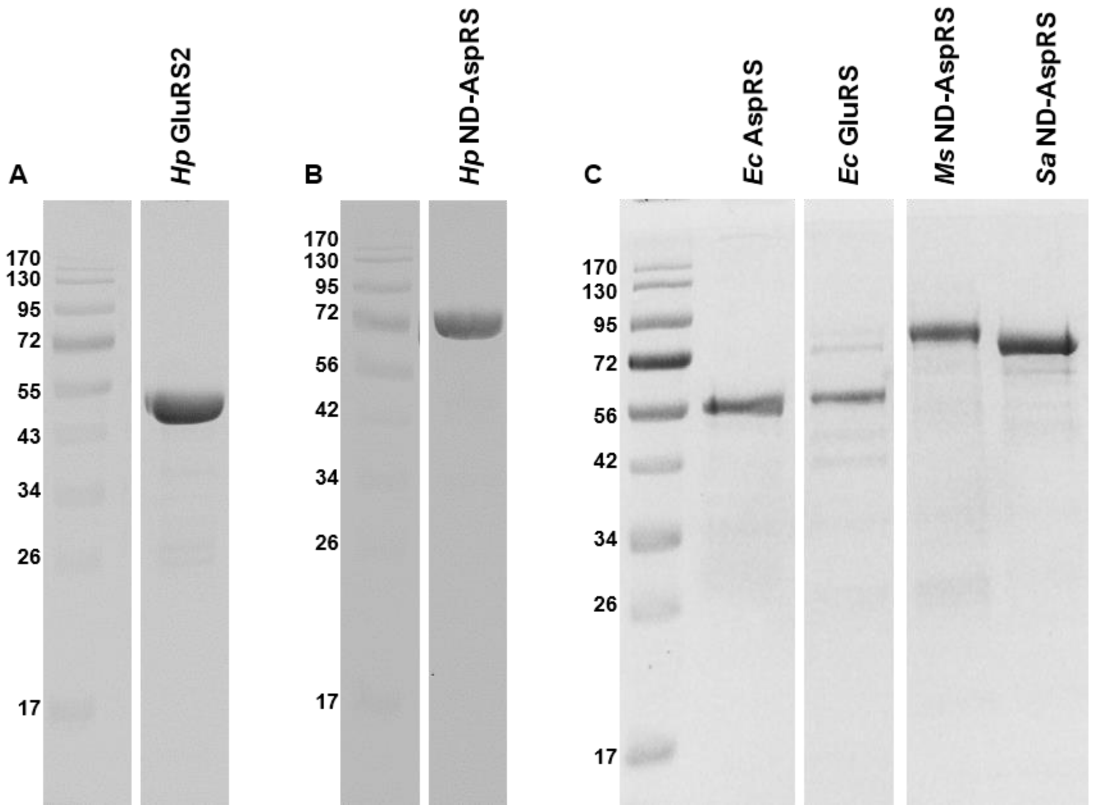


**Figure S1. SDS-PAGE gels of purified aaRSs.** Each $His_6$-tagged protein was purified to near homogeneity by cobalt affinity followed by DEAE column purification. The proteins were loaded onto an SDS-PAGE gel after this two-step purification. For clarity, intermittent lanes were removed, as indicated by the white break between different lanes. Each panel shows the results from a single gel. **(A)** SDS-PAGE analysis of *Hp* GluRS2. **(B)** SDS-PAGE analysis of *Hp* ND-AspRS. **(B)** SDS-PAGE analysis of *Ec* AspRS, GluRS, and ND-AspRS from *Ms* and *Sa.*

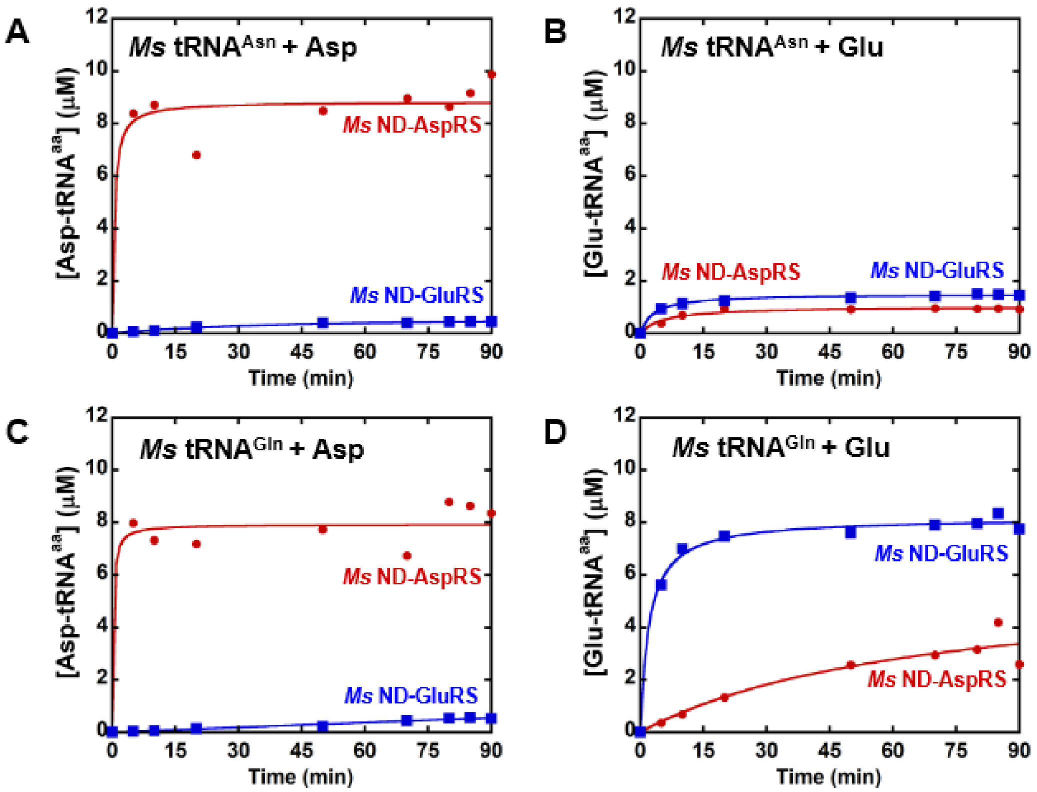


**Figure S2. Extended *M. smegmatis* tRNA$^{Asn}$ and tRNA$^{Gln}$ aminoacylation assays with *M. smegmatis* ND-AspRS and ND-GluRS with aspartate versus glutamate.** *Ms* ND-AspRS (●, 1 µM) and ND-GluRS (■, 1 µM) were tested in cross-aminoacylation assays using *Ms* tRNA$^{Asn}$ and tRNA$^{Gln}$ with aspartate and glutamate. The tRNA isoacceptor concentration in each assay was 10 µM; but each tRNA isoacceptor was contaminated with total *Ec* tRNA. **(A)** *Ms* tRNA$^{Asn}$ aminoacylated with aspartate, **(B)** *Ms* tRNA$^{Asn}$ aminoacylated with glutamate, **(C)** *Ms* tRNA$^{Gln}$ aminoacylated with aspartate, and **(D)** *Ms* tRNA$^{Gln}$ aminoacylated with glutamate.

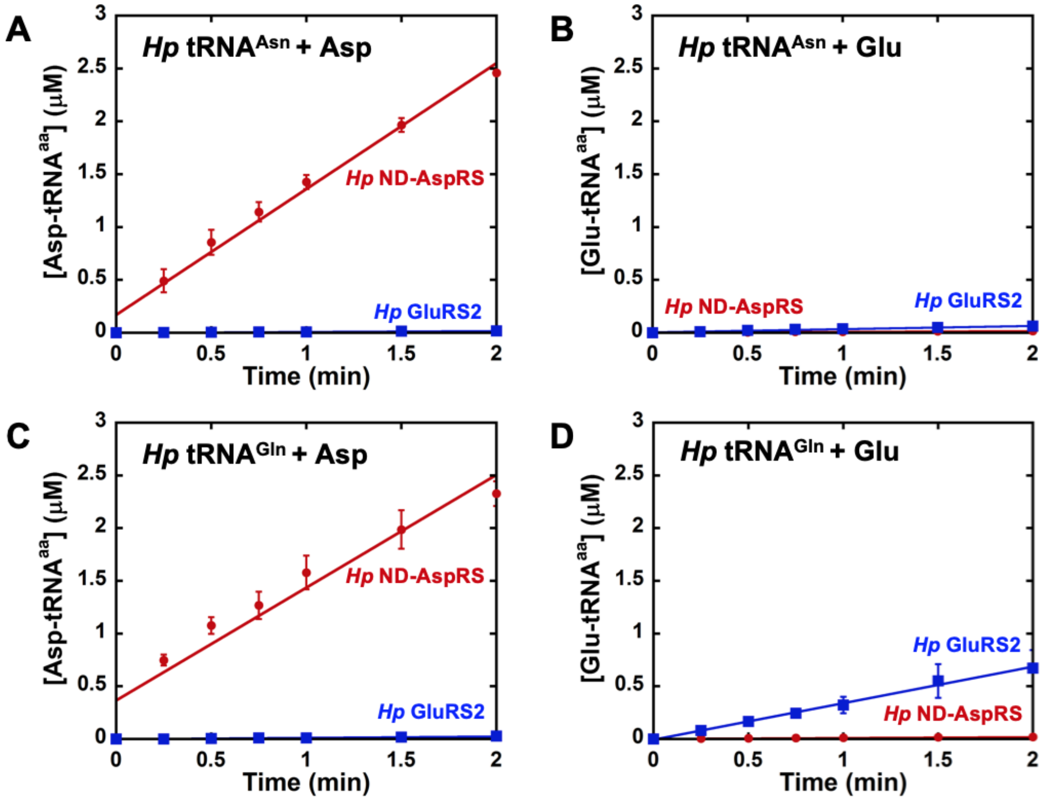


**Figure S3. *H. pylori* ND-AspRS shows unexpected aminoacylation activity with overexpressed *H. pylori* tRNA$^{Gln}$**. *Hp* ND-AspRS (●, 200 nM) and GluRS2 (■, 200 nM) were tested in cross-aminoacylation assays using *Hp* tRNA$^{Asn}$ and tRNA$^{Gln}$ with aspartate and glutamate. The tRNA isoacceptor concentration in each assay was 10 μM; however, each tRNA isoacceptor was contaminated with total *Ec* tRNA. **(A)** *Hp* tRNA$^{Asn}$ aminoacylated with aspartate, **(B)** *Hp* tRNA$^{Asn}$ aminoacylated with glutamate, **(C)** *Hp* tRNA$^{Gln}$ aminoacylated with aspartate, and **(D)** *Hp* tRNA$^{Gln}$ aminoacylated with glutamate. Error bars represent standard deviation from biological replicates in triplicate.

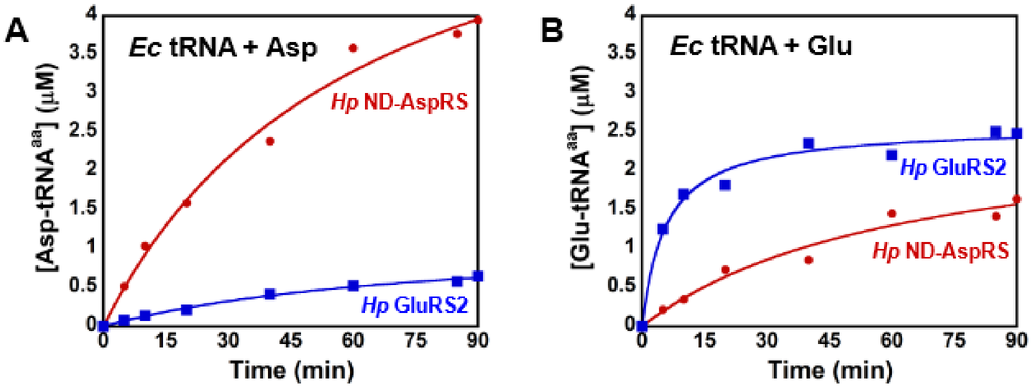


**Figure S4. Extended total *E. coli* tRNA aminoacylation assays with *H. pylori* ND-AspRS and GluRS2 with aspartate versus glutamate.** *Hp* ND-AspRS (●, 1 μM) was tested for its activity with *Ec* tRNA (50-100 μM) and aspartate versus glutamate. *Hp* GluRS2 (■, 1 μM) was also assayed for comparison. **(A)** Aminoacylation of *Ec* tRNA with aspartate. **(B)** Aminoacylation of *Ec* tRNA with glutamate.

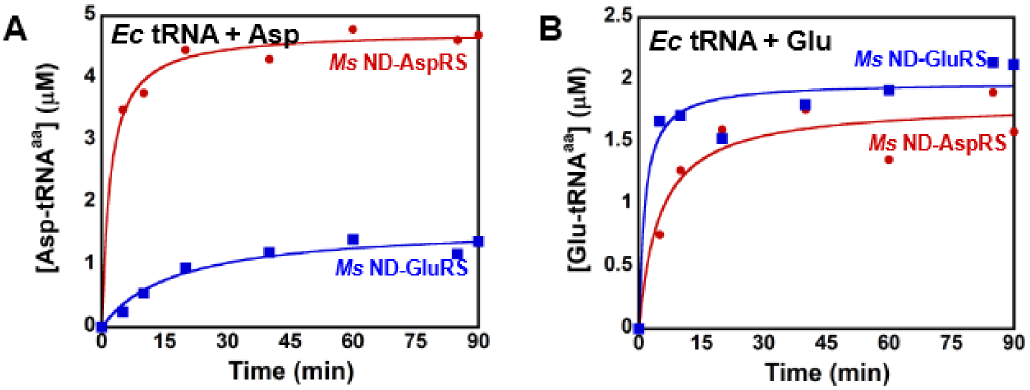


**Figure S5. Extended total *E. coli* tRNA aminoacylation assays with *M. smegmatis* ND-AspRS and ND-GluRS with aspartate versus glutamate.** *Ms* ND-AspRS (●, 1 μM) was tested for its activity with *Ec* tRNA (50-100 μM) and aspartate versus glutamate. *Ms* ND-GluRS (■, 1 μM) was also assayed for comparison. **(A)** Aminoacylation of *Ec* tRNA with aspartate. **(B)** Aminoacylation of *Ec* tRNA with glutamate.

**Table S1: The tRNA specific oligonucleotide sequences used in northern blot analysis**

| **tRNA** | **Sequence** |
|---|---|
| *H. pylori* $tRNA^{Gln}$ | CTCGGAATGCCAGGACCAA |
| *E. coli* $tRNA^{Glu}$ | CCCTGTTACCGCCGTGAAA |
| *E. coli* $tRNA^{Gln(UUG)}$ | CAGGGAATGCCGGTATCAAA |
| *E. coli* $tRNA^{Asp}$ | CCGCGACCCCCTGCGTGACA |
| *E. coli* $tRNA^{Asn}$ | CAGTGACATACGGATTAACA |
